# Prediction of response to sofosbuvir-based therapy using serum interleukin-12 and single nucleotide polymorphism of the interleukin 28B gene as predictive factors in HCV positive genotype-4 patients

**DOI:** 10.1097/MD.0000000000034125

**Published:** 2023-07-14

**Authors:** Doaa Mohamed Abdelnajid, Ahmed Y. Elmowafy, Lionel Rostaing, Marwa T. Elrakaiby

**Affiliations:** a Department of Microbiology and Immunology, Military Medical Academy, Cairo, Egypt; b Department of Microbiology and Immunology, Faculty of Pharmacy, Cairo University, Kasr Al-Aini, Cairo, Egypt; c Urology and Nephrology Center, Mansoura University, Mansoura, Egypt; d Department of Nephrology, Hemodialysis, Apheresis, and Kidney Transplantation, CHU Grenoble-Alpes, France; e Grenoble Alpes University, Grenoble, France.

**Keywords:** direct-acting antiviral agents, drug resistance, genetic polymorphism, HCV genotype 4, Hepatitis C infection, IL12, IL28B rs12979860 SNP

## Abstract

Some hepatitis-C virus patients have resistance to direct-acting-antivirals (DAAs). Genetic polymorphisms have been associated with drug resistance. This study aimed to evaluate the role of interleukin (IL)-28B gene polymorphism and IL-12 levels as predictors for a response to sofosbuvir/ribavirin (SOF/RBV) with (triple-therapy) or without (dual-therapy) Peg-alpha-interferon. 92 hepatitis C virus (HCV)/RNA (+)-patients treated with dual (n = 72) or triple (n = 20) therapy. IL28B genetic polymorphism and IL-12 level assessments. 30.4% of the patients were IL28B C/C genotype, 56.5% C/T-genotype, and 13% T/T-genotype. Mean baseline IL-12 levels were 27.5 ± 3.0 pg/mL. Rapid viral response was achieved in 86/92 patients. All patients achieved end-of-treatment virologic response. The 12- and 24-week sustained virologic responses (SVR) were achieved in 76 patients (82.6%), that is, a relapse was found in 16 patients (17.4%). 8 and 12-weeks after antiviral therapy, IL-12 levels decreased significantly, and became comparable to those of the control-group. That drop in IL-12 levels was similar across the dual- and triple-therapy patients. Finally, logistic regression analysis showed that the increase in baseline aspartate aminotransferase (AST) and T/T genotyping had an independent effect on increasing the probability a SVR failing in both dual- and triple-therapy groups (*P* = .0007 and *P* = .02, respectively). Single-nucleotide polymorphism (SNP) in IL-28B and IL-12 levels play roles as predictors in DAAs resistance.

## 1. Introduction

Infection with the hepatitis C virus (HCV) is the leading cause of chronic liver disease worldwide. Egyptians have a high prevalence of HCV genotype 4, a poor response to antiviral therapy, and an increased risk for developing liver complications.^[1]^

With the advent of direct-acting antiviral (DAA) therapies for HCV, many patients can now expect to achieve a sustained virological response (SVR) In contrast to historical treatments with pegylated alpha-interferon (Peg-IFN) and ribavirin (RBV), DAAs deliver SVR rates in the order of 90% to 95% and higher.^[2]^

It has been suggested that single-nucleotide polymorphisms (SNPs) in some cytokine genes may influence the production of the associated cytokines, which affect the host immune response in several infectious diseases. In the setting of chronic HCV infection, the production of inappropriate amounts of cytokines has been associated with persistent viremia, hepatic fibrogenesis, and even resistance to therapy.^[3]^

The cytokine profile plays an important role in treatment response to peg-IFN-α and RBV, and probably modulates the immune response against HCV. Therefore, during anti-HCV therapy, interferon (IFN)-α stimulates Th1 cells, but restrains Th2 response, suggesting that Th1 rather than Th2 cytokines may be important in eliminating the HCV infection. Besides, anti-HCV therapy is accompanied by a decreased secretion of the Th2 cytokines such as IL-4 and IL-10.^[4]^

It has been demonstrated that polymorphisms near the interleukin-28B (IL28B) gene on chromosome 19 predict the response to Peg-IFN--RBV therapy in HCV-infected patients. This SNP was identified in a genome-wide association study. IL-28 is a cytokine that exists in 2 isoforms, IL-28A and IL28B, and plays a role in the immune defense against viruses.^[5]^

Little is known about predictors of failure to achieve a SVR with DAAs. Although numerous parameters predict a poor response to Peg-IFN treatment (e.g., age, ethnicity, human immunodeficiency virus co-infection, insulin resistance, and IL28B genotype), none have been shown to be associated with a virological relapse after DAA-based therapy.^[6]^

It is well known that cytokines and chemokines play a significant role in modulating the immune response and defense against viral infections. So far, only a limited number of studies have investigated the cytokines and chemokines involved in chronic HCV infections. The expression of cytokines and chemokines in patients treated with polyethylene glycol IFN and RBV has been reported. However, the expression of serum cytokines and chemokines in response to sofosbuvir (SOF) and RBV-based treatment remain unclear.^[7]^

The aim of this study was to evaluate the role of gene polymorphism of IL28B and IL-12 expression to predict an early response to HCV treatment with SOF and/or Peg-IFN, and to assess the antiviral efficacies of dual- and triple-combination therapies.

## 2. Patients and methods

### 2.1. Patients

It is a case-control study, in which we included one-hundred patients chronically infected with HCV genotype 4. They were recruited at Al-Maadi medical complex, Cairo, Egypt. Institutional Review Board Statement: The research was approved from the ethical committee number: MI 1314 from Faculty of Pharmacy, Cairo University.

All patients that fulfilled the following criteria were enrolled, that is, men and non-pregnant women aged > 18 years, being chronically HCV-infected with genotype-4. HCV status was assessed by a serological test to detect the HCV antibody and by PCR for genotyping and quantification of HCV viral load. We excluded any patient that had one or more of the following criteria, that is, co-infection with hepatitis B virus, assessed by standard serological tests (HBs antigen); serum creatinine ≥ 2.5 mg/dL or estimated glomerular-filtration rate lower than 30 mL/min; platelet levels of ≤50,000/mm³; thyroid disease; taking amiodarone as a treatment for cardiac problems; the presence of any chronic illness; pregnancy or inability to use effective contraception for female sub-jects; and if any data showed a HCV genotype other than genotype 4.

However, 8 patients were excluded from further analysis due to screening failures, discontinuation of therapy (non-virologic failures, e.g., due to severe side-effects).

HCV-patients were divided into 2 main groups according to their received regimen: the dual-therapy group included 72 patients that had received SOF plus RBV. The 20 patients in the triple-therapy group received Peg-IFN plus SOF--RBV. Furthermore, all patients were divided into 2 subgroups according to their response to treatment, that is, responders and non- responders. The decision to pre-scribe the dual or triple therapy was based on the degree of hepatic fibrosis, that is, the triple therapy was given to patients with advanced fibrosis. Dual therapy consisted in SOF given as a 400-mg pill taken once daily in combination with 600 mg to 1400 mg of RBV per day according to body weight. In triple therapy we added Peg-Interferon at 180 mcg per week. Treatment duration was 12 weeks for IFN-naïve patients (triple therapy) and 24 weeks for patients with history of previous IFN treatment (dual therapy).

Response to treatment was determined according to the results of HCV PCR assessed at different time-points. Rapid viral response (RVR) was defined by viral clearance within 3 weeks of initiating treatment. End-of-treatment viral response was de-fined as viral clearance at the end of treatment. Twelve-week sustained viral response (12-SVR) was defined as viral clearance at up to 12 weeks after treatment completion. Finally, 24-week sustained viral response (24-SVR) was defined as viral clearance up to 24 weeks after treatment completion.

Data collection: Data sheets from the included patients were revised for patient age, gender, liver- or kidney-transplant status, and baseline laboratory investigations, which included HCV PCR, a complete blood picture, liver-function tests (albumin, bilirubin, International Normalized Ratio [INR], alanine aminotransferase [ALT], and aspartate aminotransferase [AST]), liver biopsy, clinical and radiological findings (including ascites, cirrhosis, splenomegaly and degree of fibrosis as detected by Metavir score). Individual fibrosis stage was documented as stage 0 to 1, stage 2, or stage 3 to 4 (i.e., absence or minimal fibrosis, moderate fibrosis, or advanced fibrosis/presence of cirrhosis according to the Metavir scoring system F1-4).^[8]^

Laboratory investigations were repeated routinely during and after treatment and when needed. HCV PCR was done at baseline, 4-week after treatment initiation, at the end of treatment, and at 12 and 24 weeks after treatment completion.

Apart from HCV-positive patients we included 30 healthy persons, that is, the control group.

The study complies with CONSORT 2010 rules.

### 2.2. Genetic and cytokine assessments

Blood samples from HCV Patients were withdrawn at 3 different time points: prior to treatment initiation, at week 8 after starting treatment, and at month-3 after treatment completion. Samples from the control group were obtained at 1 time point only. When the samples were collected, after 1 hour, they were centrifuged to avoid RNA breakdown. The separated serum was then stored at −70°C.

SNP genotyping for IL28B (rs12979860): DNA collection and extraction were as follows. Before treatment, blood samples were collected from all patients into EDTA tubes, which were then used for DNA extraction using a G-spin total-DNA extraction kit (Intron Biotechnology, Korea) according to the manufacturer instructions. DNA purity was assessed using Nano Drop (Clini Lab Cairo, Egypt). With regards to genotyping of SNP IL28B TaqMan SNP IL28B Real-Time PCR Genotyping of rs12979860 variant was conducted using custom-designed primers and probes purchased from Integrated DNA Technology (IDT) Company, USA. (Forward primer: rs12979860_F GCCTGTCGTGTACTGAACCA; re-verse primer: rs12979860_R GCGCGGAGTGCAATTCAAC; probes: rs12979860_V VIC-TGGTTCGCGCCTTC-NFQ and rs12979860_M FAM-CTGGTTCACGCCTTC-NFQ).

All reactions were set up using 1µl of isolated gDNA and a TaqMan Genotyping Master Mix: genotyping was done by a Step One Plus instrument (Life Technology, Carlsbad, California, USA) performed in Clinilab Cairo, Egypt. Temperature cycling profiles were as follows; an initial denaturation at 95ºC for 10 minutes, 40 cycles of denaturation of DNA at 95ºC for 10 seconds, 5 seconds of annealing of primers at 58ºC and extension at 72ºC for 20 seconds. The detection of end-point fluorescent products was monitored once every cycle.

Analysis of IL-12 levels: A quantitative sandwich enzyme-linked immuno-assay technique, based on an IL-12 kit (Sun Red Biotechnology Company, Shanghai, China) was used for the quantitative measurement of the IL-12 in patients’ serum. A best-fit curve was drawn by plotting the net optical density (average of duplicate readings minus blank OD) of the standards versus their concentrations: the best-fit line was determined, and IL-12 concentration of each sample was estimated from the curve.

### 2.3. Statistical analyses

The sample size was calculated by using the G-power program with α. Error = 0.05 and power 80% and was equal to a minimum of 80 patients.

Recorded data were analyzed using the statistical package for social sciences, version 23.0 (SPSS Inc., Chicago, IL). Student *t* test was used when comparing between 2 means. The paired sample *t* test of significance was used when comparing between related samples. A one-way analysis of variance was used to compare between more than 2 means. The post hoc test: least significant difference, was used for multiple comparisons between different variables. The chi-squared (×2) test was used to compare proportions between qualitative parameters. Logistic regression was used to predict the presence or absence of an outcome based on independent variables. Receiver operating characteristic (ROC curve) analysis was used to detect the accuracy of different markers.

## 3. Results

### 3.1. Demographics and baseline characteristics

This study included 92 HCV patients and 30 healthy, age, and gender-matched controls. All hepatitis C patients were presented by either accidentally discovered positive HCV serology or development of hepatic manifestations as abnormal liver/spleen imaging or ascites. Six patients received renal transplantation and the original kidney disease was not related to extra-hepatic manifestations of HCV. There were significant differences between the HCV group and healthy control group regarding serum albumin (control vs study group: 4.2 ± 0.6 vs 3.8 ± 0.5 g/dL; *P* = .001), ALT (control vs study: 35.53 ± 12.69 vs 52.57 ± 34.56 IU/L; *P* = .009), and AST (control vs study: 27.8 ± 10 vs 56.4 ± 34.4 IU/L; *P* = .001). In contrast, platelets (control vs study: 254.7 ± 80.0 vs 197.1 ± 66.0 × 103/mm^3^; *P* < .001) and total leucocyte count (control vs study: 6.63 ± 1.57 vs 5.56 ± 1.69 G/L; *P* = .003) were significantly lower among the HCV group (Table 1).

**Table 1:** Baseline and follow-up laboratory investigations between dual- and triple-therapy groups.

Variable	Dual therapy (n = 72)	Triple therapy (n = 20)	*P* value
	Mean ± SD	Mean ± SD	
Albumin (g/dL)	3.99 ± 0.5	3.9 ± 0.3	.8
Total bilirubin (mg/dL)	0.8 ± 0.3	0.6 ± 0.2	*.001*
ALT (IU/L)	53.3 ± 36.4	49.9 ± 27.4	.69
AST (IU/L)	57.1 ± 34.4	54.0 ± 35.1	.72
INR	1.1 ± 0.2	1.0 ± 0.1	.08
HCV RNA (PCR) (IU/mL) Median (min, max)	818,500 (3453, 1878283)	287,500 (2343, 2123458)	.06
*Hemoglobin (g/dL*)			
Baseline	13.1 ± 1.3	13.1 ± 1.1	.92
After 8 wk (W8) of treatment	10.8 ± 1.2	10.6 ± 1.0	.53
Difference between BL and W8	−2.2 ± 1.4	−3.0 ± 1.8	*.025*
*Platelets (×10*^*3*^*/mm*^*3*^)			
Initial	190.4 ± 63.6	221.4 ± 70.3	.06
After 8 wk of treatment	173.8 ± 57.0	183.2 ± 58.6	.52
Difference between BL and W8	−21.9 ± 37.1	−18.9 ± 92.3	.823
*TLC (×10*^*3*^*/mm*^*3*^)			
Initial	5.6 ± 1.7	5.7 ± 1.7	.82
After 8 wk of treatment	5.5 ± 2.0	4.9 ± 1.4	.15
Difference between BL and W8	−0.5 ± 2.3	1.0 ± 2.8	*.02*

ALT = alanine aminotransferase, AST = aspartate aminotransferase, BL = baseline, Hb = hemoglobin, HCV = hepatitis C virus, INR = international normalized ratio, SD = standard deviation, TLC = total leucocyte count, W = week.

All HCV patients had a positive viral load, that is, mean HCV PCR was 1981,495.0 ± 4597,64.0 IU/mL. A total of 78.2% of patients received dual therapy in the form of SOF and RBV, while 21.8% also received in addition Peg-IFN. About 30.4% of the included patients were the IL28B C/C genotype, 56.5% were the C/T genotype, and 13% the T/T genotype. Mean values of basal IL-12 were 27.5 ± 3.0.

Comparisons between the 2 groups showed no significant difference regarding the prevalence of liver transplantation, renal transplantation, splenomegaly, ascites, cirrhosis, or fibrosis stage. There were significantly more naïve patients within the triple therapy group compared to the double-therapy group, which was consistent with the drug-assignment criteria (*P* < .0001). There were no statistically significant differences between the groups regarding RBV-dose modification, adding recombinant erythropoietin, or the development of hepatocellular carcinoma (Table 2). One patient developed hepatocellular carcinoma. His baseline fibroscan was F3. Hepatic imaging showed presence of enlarged liver with cirrhotic features. Baseline serum bilirubin (0.6 mg/dL), ALT (28 IU/L), and AST (16 IU/L) were within normal ranges. Serum albumin was slightly low (3.1 g/dL) but there was no ascites or edema. During treatment (dual therapy), there was no specific events as liver enzymes were within normal ranges, he achieved RVR and 12-week SVR successfully. 3-months after treatment completion, he developed ascites, lower limb edema and jaundice. Liver enzymes elevated (ALT:78IU/L; AST: 60 IU/L). Serum albumin was 2.1 (g/dL) and serum bilirubin was (1.9 mg/dL). Ultrasound revealed focal lesion in segment IV of the liver. Diagnosis of HCC was confirmed by Triphasic CT.

**Table 2:** Comparison between dual and triple therapies regarding response to treatment.

	Dual therapy	Triple therapy	*P* value
Rapid virological response	67 (93.1%)	19 (95%)	.76
12-wk sustained viral response	58 (80.6%)	18 (90%)	.32
24-wk sustained viral response	58 (80.6%)	18 (90%)	.32

Baseline bilirubin was significantly higher in the double-therapy group compared to the triple-therapy group (*P* = .001) but no significant difference for baseline albumin, ALT, AST, INR, or HCV RNA. There was no statistical difference between the 2 groups regarding hemoglobin level and leukocytes and platelet counts at baseline and after 8 weeks of treatment. However, the drop-in hemoglobin (*P* = .025) and platelet numbers (*P* = .02) was greater among the triple-therapy group (Table 3).

**Table 3:** Interleukin-12 between health controls and dual/triple therapy at different time point.

	Control group	Baseline	After 8 wk	After 12 wk	*P* value
Dual therapy	10.35 ± 1.37	27.50 ± 2.98 (a)	10.95 ± 1.33 (b)	8.89 ± 0.93 (a, b, c)	*<.001 (F*)
Triple therapy	10.35 ± 1.37	27.50 ± 2.98 (a)	7.18 ± 0.75 (a, b)	8.07 ± 0.95 (a, b, c)	*<.001 (F*)
*P* value between dual and triple therapy		1.000 (t)	*<.001 (t*)	*.008 (t*)	

(F) Analysis of variance (ANOVA) test; (t) Student *t* test; Level of significance < 0.05; (a, b, c) post hoc analysis; (a) Significance against control < 0.05; (b) Significance against baseline < 0.05; (c) Significance against after 8 wk < 0.05.

A RVR was achieved in 86 of 92 patients.

### 3.2. Virological response of hepatitis C virus genotype 4 (HCV-4) infected patients after dual and triple Sovaldi combination therapy

All patients achieved an end-of-treatment virologic response. The 12- and 24-week SVRs were achieved in 76 patients (82.6%), that is, a relapse was found in 16 patients (17.4%). All the relapses occurred within 3 months after stopping antiviral treatment.

There were no statistical differences between the dual- and triple-therapy groups regarding RVR, end-of-treatment virologic response, and the 12- and 24-week SVR (Table 4).

**Table 4:** Interleukin-12 levels and interleukin 28B genotyping as predictors for a non-response.

	Responders (n = 76)	Non-responders (n = 16)	*P* value
Interleukin-12, mean ± SD			
*Total cohort*			
Before treatment	28.6 ± 2.4	24.1 ± 1.5	*<.001*
After 8 wk	9.7 ± 2.2	10.3 ± 1.6	.38
After 12 wk	8.5 ± 1.0	9.0 ± 0.3	.08
*Dual therapy*			
Before treatment	28.3 ± 2.6	24.3 ± 1.8	*<.001*
After 8 wk	11.1 ± 1.3	10.6 ± 1.5	.25
After 12 wk	8.8 ± 1.0	9.1 ± 0.9	.46
*Triple therapy*			
Before treatment	28.9 ± 2.5	23.1 ± 0.1	*.005*
After 8 wk	7.1 ± 0.7	8.0 ± 0.6	.10
After 12 wk	8.0 ± 0.9	9.0 ± 0.9	.14
Interleukin-28B genotyping, No. (%)			
*Total cohort*			
C/C	25 (32.9%)	3 (10.7%)	.26
C/T	46 (60.5%)	6 (11.5%)	.09
T/T	5 (6.6%)	7 (58.3%)	*<.001*
*Dual therapy*			
C/C	16 (21.1%)	2 (12.5%)	.30
C/T	38 (50%)	5 (31.3%)	*.04*
T/T	4 (5.3%)	7 (43.8%)	*<.001*
*Triple therapy*			
C/C	9 (11.8%)	1 (6.3%)	1.000
C/T	8 (10.5%)	1 (6.3%)	.88
T/T	1 (1.3%)	0 (0.0%)	.73

### 3.3. Correlation of treatment outcome with IL-12 serum levels

Baseline IL-12 levels were significantly lower in the control group than in the HCV group, that is, 10.4 ± 1.37 versus 27.5 ± 3.0 pg/mL (*P* < .05). Regarding IL-12 levels there were no statistical difference at baseline between dual and triple therapy patients. However, after 8 and 12 weeks, IL-12 levels decreased significantly, and became com-parable to the control group. That drop in IL-12 levels was similar across the dual and triple therapy patients, that is, 8.9 ± 0.9 pg/mL and 8.1 ± 1.0 pg/mL at 12 weeks in dual and triple therapy patients respectively (*P* < .001).

### 3.4. Frequencies of IL28B rs12979860 genotypes and their association with different treatment outcome

From Taq-man probe RT-PCR SNP genotyping we found that IL28B C/C variant frequency in patients in the triple-therapy group was 10/20 (50%), whereas it was 18/72 (25%) in the dual-therapy group (*P* = .03). The incidence of IL28B C/T variant was 9/20 (45%) and 43/72 (59.7%) in the triple- and dual-therapy groups, respectively. The frequency of IL28B rs12979860 T/T allele was 1/20 (5%) and 11/72 (15.3%) (37.5%) in the triple- and dual-therapy groups, respectively (ns).

Serum IL-12 levels were statistically higher among responders than non-responders at baseline in both cohorts (*P* < .001; dual therapy: *P* < .001; triple therapy: *P* = .005). However, IL-12 levels at 8 after therapy were comparable in the total cohort (responders vs non-responders: 9.7 ± 2.2 vs 10.3 ± .16; *P* = .38), in dual therapy group (responders vs non-responders: 11.1 ± 1.3 vs 10.6 ± 1.5; *P* = .25) and in triple therapy group (responders vs non-responders: 7.1 ± 0.7 vs 8 ± 0.6; *P* = .1). Also, there were no statistically significant differences between responders and non-responders regarding IL-12 levels after 12 weeks in total cohort (responders vs non-responders: 8.5 ± 1 vs 9 ± 0.3; *P* = .08), in dual therapy group (responders vs non-responders: 8.8 ± 1 vs 9.1 ± 0.9; *P* = .0.46) and in triple therapy group (responders vs non-responders: 8 ± 0.9 vs 9 ± 0.9; *P* = .14).

Regarding IL28B genotyping, both responders and non-responders were comparable regarding C/C and C/T genotype expression, whereas T/T expression was significantly higher in non-responders (*P* < .001). In the dual-therapy group, responders and non- responders were comparable regarding C/C genotype expression. C/T genotype expression was significantly higher among responders (*P* = .043), whereas T/T expression was significantly higher among non- responders (*P* < .001). In the triple-therapy group, both responders and non-responders were comparable regarding IL28B genotype expression as C/C expression was 11.8% in responders versus 6.3% in non-responders (*P* = 1.00), C/T expression was 10.5% in responders versus 6.3% in no-responders (*P* = .88) and T/T expression was 1.3% in responders versus 0% in non- responders (*P* = .73) (Table 5).

**Table 5:** Logistic regression model for the factors affecting failure of a SVR in both groups using the Backward method.

Independent variables	Coefficient	Std. Error	Wald	*P* value	Sig.
(Constant)	55.7941				
Age	---	---	---	---	NS
Sex	---	---	---	---	NS
Liver transplantation	---	---	---	---	NS
Renal transplantation	---	---	---	---	NS
Splenomegaly	---	---	---	---	NS
Ascites	---	---	---	---	NS
Cirrhotic liver	---	---	---	---	NS
Fibro-scan (fibrosis stage)	---	---	---	---	NS
Antiviral drug history	---	---	---	---	NS
Albumin (g/dL)	---	---	---	---	NS
Total bilirubin (mg/dL)	---	---	---	---	NS
ALT (µ/L)	---	---	---	---	NS
AST (µ/L)	0.15096	0.044642	11.4344	.0007**	S
INR	---	---	---	---	NS
Hb (g/dL)	---	---	---	---	NS
Platelets (10^3^/µL)	---	---	---	---	NS
TLC (10^3^/µL)	---	---	---	---	NS
HCV RNA (PCR) (iu/ml)	---	---	---	---	NS
SNP IL-28b genotyping (T/T)	4.15304	1.81992	5.2075	.0225*	S
SNP IL-28b genotyping (C/T)	---	---	---	---	NS
SNP IL-28b genotyping (C/C)	---	---	---	---	NS

ALT = alanine aminotransferase, AST = aspartate aminotransferase, HCV = hepatitis C virus, IL = interleukin, SNP = single nucleotide polymorphism, SVR = sustained virological response.

--- excluded from the model if (*P* value > 0.1).

### 3.5. Predictors for response to DAAs

All predictive values were entered in logistic regression analysis using Backward method to be adjusted for the confounders and to adjust SNP IL28B against other fac-tors. Only 2 factors had statistically significant effect on the probability of SVR failure, that is, the increase in baseline AST and T/T genotyping had an independent effect on increasing the probability a SVR failing in both groups (*P* = .0007 and *P* = .02, respectively). The prevalence of T/T genotype was increased in patients with a SVR failure (non-responders), whereas C/C and C/T prevalence were increased in patients that achieved a SVR (responders) (Table 6).

**Table 6:** Prediction of response to triple and dual therapies using IL-12.

Serum IL-12 level	cutoff	Sensitivity	Specificity	PPV	NPV	AUC [95% CI]
Before treatment	≥25.5 pg/mL	93.8%	77.8%	55.6%	97.7%	0.914 (0.822–0.968)

AUC = area under the curve, IL = interleukin, NPV = negative predictive value, PPV = positive predictive value, ROC = receiver operating characteristic, SE = standard error.

**Figure 1.**
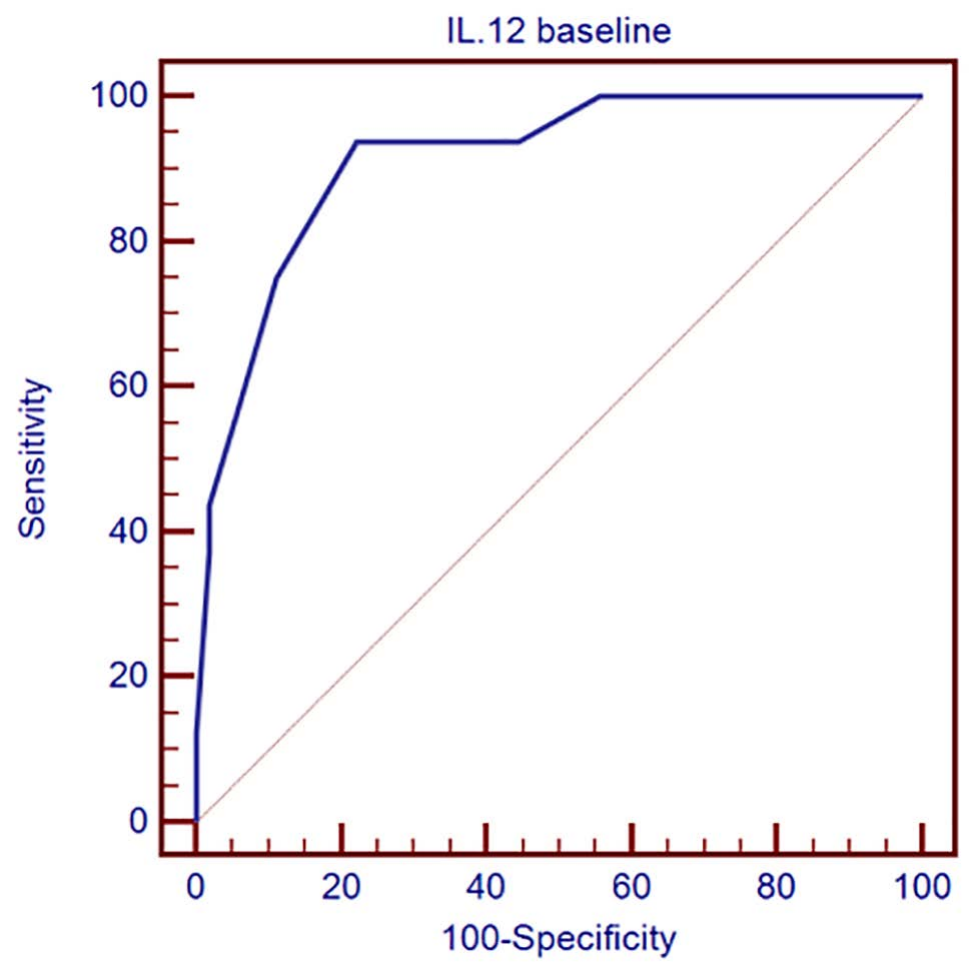
ROC curve for prediction of response to DAA therapy using IL12 level at baseline in the HCV group. DAA = direct-acting-antivirals, HCV = hepatitis C virus, IL = interleukin, ROC = receiver operating characteristic.

Finally, using ROC curve for prediction of response to DAA therapy using IL-12 level at baseline in the HCV group with a cutoff value of 25.5 pg/mL according to ROC curve, IL-12 had 93.8% sensitivity and 77.8% specificity to predict a response to direct acting antivirals (Fig. 1).

## 4. Discussion

In our study we found that when HCV RNA positive patients are given SOF/RBV therapy with or without Peg-interferon RVR is achieved in 93.5%, whereas an end-of-treatment virologic response was achieved in 100%. In addition, the 12- and 24-week SVRs were achieved in 76 patients (82.6%), that is, a relapse was found in 16 patients (17.4%).

In agreement with this result, in the general population Zuezem et al, reported a 99% RVR among HCV-positive genotype 2 or 3 patients when treated with SOF--RBV^[9]^; Other studies by Gane et al, & Charlton et al, obtained similar results.^[10,11]^

In our study we observed a SVR at 12- and 24-weeks post-treatment in 82.6% of patients, with a relapse in 16 cases. The relapse developed within the first 3 months after completing treatment. A better SVR at 12- and 24-weeks after treatment was achieved by Zuezem et al, (93%): this difference may be related to the virus genotype, as most of the Egyptian population are genotype 4 whereas Zuezem et al patients were mostly genotype 2 and 3.^[9]^ A SVR of (100%) at 24 weeks was reported by Gane et al, regardless of the virus genotype, and was achieved with SOF--RBV but also with SOF--RBV--IFN.^[10]^ A lower SVR rates (70%) were reported in other studies but some of these studies were conducted on liver transplant or immunocompromised patients.^[11–14]^ Osinusi et al, reported that a 24-week regimen of SOF and weight-based or low-dose RBV resulted in SVR24 rates of 68% and 48%, respectively.^[15]^

HCV PCR clearance and achieving SVR successfully had positive impact on the extrahepatic HCV manifestations. Rossi et al, stated that the rates of these non-liver diseases were largely reduced for those who were cured with alpha-interferon-based treatments. Early HCV treatments could provide many benefits in the prevention of various HCV complications beyond liver disease.^[16]^

Sasso et al, concluded in a study in which that regardless of the degree of liver disease and CV risk factors, HCV eradication by DAAs permitted a large reduction of MACEs in the prediabetic group and is therefore a main goal.^[17]^ Also, Adenolfi et al, reported in their multicenter study that HCV clearance has advantages both in prognostic and clinical terms and has a significant impact on public health with savings on healthcare costs. These data indicate that the treatment of HCV infection must be done not only to reduce the risk of progression/complications of liver disease, but also to reduce the risks of a serious extrahepatic HCV-related complication such as CV disease.^[18]^

Adenolfi et al, in another study proposed that clearance HCV clearance (PCR) and achieving SVR was associated with decreased incidence of type 2 diabetes mellitus as HCV induces disturbance in glucose homeostasis.^[19]^

There was a significant difference between the HCV group and the healthy control group regarding albumin, ALT, and AST (*P* = .001, 0.009, 0.001, respectively) at baseline. Hypoalbuminemia is a common sign of liver fibrosis after hepatitis and is as-sociated with an increased risk of mortality and it was detected in 20.9% of patients.^[15,20,21]^

Sugimoto et al, and Austria et al, reported that HCV patients had impaired synthetic liver function with elevated liver enzymes, like the results from our study.^[22,23]^ Both our groups were comparable regarding serum bilirubin. Dascal et al stated that hyperbilirubinemia is not a feature of HCV infection, and its presence reflects ex-acerbation of liver disease.^[24]^ However, Shaker et al found that hyperbilirubinemia was a consistent feature of HCV infection.^[25]^

Platelet count was significantly lower among our HCV group (*P* < .001). This agrees with other studies, for example, Huang et al,^[26]^ Abdullah et al,^[27]^ Wrotkowska et al reported the presence of thrombocytopenia in up to 70% of HCV patients and was one of the commonest hematological abnormalities with HCV.^[21]^ Hemoglobin levels were higher among our HCV group. This is consistent with Abdullah et al, & Elnaggar et al, who did not find a significant effect of HCV per se on hemoglobin.^[27,28]^

Our dual and triple groups were comparable regarding baseline laboratory findings. Comparisons between SOF--RBV (with and without IFN) have been evaluated in several studies and both groups similarly were comparable in baseline characteristics.^[29–33]^

In the current study, only 1 patient developed HCC after DAA treatment. This patient was in dual therapy group. It has been demonstrated previously that SVR achievement after DAAs has no impact on onset of HCC; however, HCV clearance de-creases the chance to develop HCC.^[34,35]^ Moreover, Rinaldi et al, found that SOF-based DAAs regimen without RBV was associated with higher incidence of HCC and RBV had protective effect against HCC onset.^[36]^

We found no statistical difference between the dual and triple groups at baseline and during treatment regarding levels of hemoglobin, or leucocyte and platelet counts. However, the effect of treatment on hemoglobin and leucocyte count was greater among the triple-therapy group during treatment with no statistically significant differences. In agreement with our study, Foster et al proposed that a numerically greater proportion of patients receiving SOF--RBV plus IFN for 12 weeks had grade 3 and 4 laboratory abnormalities compared to patients receiving SOF--RBV for 16 or 24 weeks.^[30]^ The median reduction in hemoglobin level at the end of treatment was 2.6 g/dL among patients receiving SOF--RBV plus IFN compared to 2.0 g/dL among patients receiving SOF--RBV for 16 weeks. In a study where HCV/RNA (+) patients were treated with SOF plus RBV with or without peginterferon-alfa for 12 weeks anemia and leucopenia occurred.^[25]^ In addition, hematologic abnormalities were more prominent among the triple-therapy group in other studies, for example, Satsangi et al,^[32]^ and Wei et al^[33]^ Adding IFN to other DAAs was associated with anemia and leucopenia in other studies.^[37,38]^ In contrast to our study, Ahmed et al found no significant effect of adding IFN to a SOF--RBV regimen on hemoglobin and leucocyte levels.^[31]^

In our triple-therapy group, the drop of HCV RNA was greater: mean values of HCV RNA were lower among the triple-therapy group within 4 weeks of treatment, that is, (354.16 ± 1444.84 IU/mL, 27.5 ± 122.98 IU/mL) for double and triple therapy respectively. Foster et al proposed that reduction of viral load was faster in the triple-therapy group than in the double-therapy group.^[30]^ In contrast to our study, Ab-bas et al found no difference between the dual and triple therapies regarding response to treatment.^[29]^

Regarding treatment efficacy, both groups were comparable regarding RVRs, 12-and 24-week SVRs. Rates of RVR ranged from 93% in the dual-therapy group to 95% in the triple therapy. The sustained viral response ranged from 81% with a dual therapy to 90% with the triple therapy. In agreement with the current study, Wei et al found comparable SVR rates among both groups (dual vs triple) with different genotypes.^[33]^ Abbas et al found RVR was greater among the triple-therapy group than with a dual therapy, although the difference was not significant.^[29]^

We found similar SVR with triple and dual therapy (90% and 80.6%, respectively), whereas Ahmed et al reported higher SVRs with a triple rather than a dual therapy (94%--83%).^[31]^ Øvrehus et al, showed higher SVR rates when IFN was added.^[39]^ Foster et al also showed that RVR and SVR were significantly higher among the triple-therapy group.^[30]^

In the VALENCE trial, SOF--RBV therapy was given to treatment-naïve and previously treated genotype-2 and genotype-3 HCV patients. Amongst HCV genotype-2 SVR12 and SVR24 response rates were 93%; in HCV genotype-3 SVR12 and SVR24 response rates were 85% and 82.4% respectively.^[9]^ In the phase II LONESTAR-2 trial, 12 weeks of SOF--RBV plus IFN was given: of the 24 patients with genotype-3 HCV, 20 (83%) achieved a 12-week SVR. There was no difference in response between patients with and without cirrhosis.^[40]^ The variable SVR rates between these different studies could be caused by the different HCV genotypes, but also to racial differences and interpersonal genetic variabilities.

The Taqman probe, RT-PCR SNP genotyping of IL28B rs12979860, was assessed in all patients; C/T variant expression had the highest frequency in both groups. Similarly, Pol et al reported that the C/T variant had the highest prevalence^[41]^; however, Wei et al, found in a Chinese population that the C/C variant had the highest prevalence.^[32]^ Also, Shaker et al proposed that the C/C variant had the highest prevalence among HCV-positive Egyptian patients,^[25]^ whereas Elwan et al reported that, among the Egyptian population, the C/C variant had the lowest prevalence among HCV-positive patients.^[42]^ Salem et al showed that the overall frequency of IL28B genotypes was 100% for genotype C/T.^[43]^ Similar findings have been noted by others.^[44]^ In our study, the C/C variant was more frequent among the triple-therapy group than the dual-therapy group (*P* = .03) whereas both groups were comparable regarding the expression of the C/T and T/T variants. In contrast, Foster et al found a semi-equal distribution of these variants between the different groups.^[30]^ Wei et al also showed comparable distribution of genetic variants between both groups,^[32]^ although Øvrehus et al found higher C/C expression among the dual-therapy group.^[39]^

On assessing the relationship between SNP genotyping of IL28B rs12979860 and response to treatment in total patients, there was no statistical difference between responders and non-responders regarding the C/C variant and C/T variant expressions. In contrast, T/T-variant expression was statistically higher among non-responders (*P* < .001), which reflects that the T/T variant is considered a risk factor for a relapse after a primary response to anti-viral treatment. In agreement with our results Foster et al reported that the T/T variant was associated with the incidence of a relapse but in our results the C/T variant also had the same association.^[30]^ Meuller et al confirmed the favorable association of the IL28B rs12979860 C/C genotype with a response to antiviral treatment and a SVR.^[45]^ There is increasing evidence that the IL28B polymorphism is a strong predictor of a SVR in patients treated for recurrent HCV after liver transplantation.^[46]^ Patients with a C/C genotype either at baseline or in the donor liver had significantly higher response rates to IFN therapy after liver transplant than the non-C/C genotypes, whereas the donor-liver C/C genotype was more predictive of a better outcome than the recipient.^[47]^ Similarly, as patients without a transplant, the IL28B C/C genotype in transplant recipients was associated with high-er SVR rates after anti-viral treatment compared to those with the non-C/C genotype.^[47]^

In the dual-therapy group, the T/T variant was also statistically associated with the incidence of a relapse (*P* < .001) whereas the C/T variant had a statistically higher frequency among responders (*P* = .043). This agrees with the findings of Foster et al where T/T-variant expression was increased among relapse receiving a dual therapy but, in contrast to our study, the C/T variant was also increased among non-responders.^[30]^ However, Mohamad et al found no statistical correlation between SNPs and IL28B and a response to SOF-based double and triple therapy.^[48]^ In the tri-ple-therapy group, there was no statistical difference between responders and non-responders regarding IL28B variants. Similar to our results, Foster et al also did not find a correlation between IL28B variants and a response to treatment among their triple-therapy group.^[30]^ However, Pol et al reported a higher relapse rate among T/T variants and a lower relapse rate among C/T variants, whereas no relapse rate was found among C/C variants in HCV patients with genotype-1 who received a triple therapy.^[41]^ In another study, a non-C/C IL28B subtype was associated with a reduced SVR-12 in patients with HCV genotype-1 treated with SOF--RBV plus IFN versus SOF--RBV only.^[33]^

SNPs in rs12979860 C/T, which are located upstream of the IL28B gene (encoding for lambda or type III IFNs), have been reported to be as-sociated with both spontaneous clearance and response to IFN-α--RBV therapy in individuals infected with HCV.^[49–51]^ In a study by Mohamad et al, the C/C variant was associated with a good outcome in HCV patients that received IFN therapy, but not DAAs.^[48]^ Another Egyptian study revealed that patients with the IL28B C/T-genotype achieved considerably higher SVRs (62.5%) compared to C/C (30%), and T/T patients (7.5%): polymorphism IL28B was an independent predictor of a SVR with IFN--RBV in Egyptian patients with HCV genotype 4.^[52]^

Regarding IL-12 levels, in our study, serum expression of IL-12 in HCV-chronic liver disease was significantly elevated compared to non-diseased individuals, and the level was enhanced with disease progression. This suggests that a strong pro-inflammatory cytokine response could play an important role in the development of hepatic injury in patients with chronic HCV infection and, therefore, apart from contributing to viral clearance, this polarized immunological profile may contribute to the pathogenesis of liver disease.^[53]^ Similar findings were reported by El-Emshaty et al as IL-12 was significantly higher among the HCV group than the disease-free group, and IL-12 also increased significantly with disease progression.^[54]^ In another Egyptian study, IL-12 serum concentrations were significantly higher in patients with liver cirrhosis when compared to healthy individuals (*P* < .001).^[55]^

In all our HCV/RNA positive patients, serum IL-12 levels decreased significantly after initiating treatment, in both dual- and triple-therapy groups. Similarly, Xu et al reported a decrease of IL-12 after treatment with SOF--RBV.^[7]^ In both dual and triple groups, we observed a statistically significant difference between baseline IL-12 levels and those found after 8 weeks of treatment, and also between baseline IL-12 levels and those found 3 months after treatment completion. Regarding IL-12 levels, in dual therapy group, there was a statistically significant difference between levels observed after 8 weeks of treatment and those found 3 months after treatment completion, while in triple therapy, comparable IL-12 levels were found at both time-points. Similar findings were reported by El-Hendawy et al,^[55]^ and agrees with the findings of Carlin et al.^[56]^

Logistic regression analysis was performed to study the independent predictors for treatment failure. We found that only AST levels at baseline and the T/T variant of the IL28B gene were independently associated with treatment failure. In contrast, other demographic data and clinical and laboratory data did not show any statistical associations as predictors for treatment failure. The value of SNP IL-28 as a predictor for treatment response has been previously analyzed. Degasperi et al considered that SNPs in the IL28B gene are the most important predictor for treatment response.^[57]^

In contrast to our study, Osinusi et al found that the risk of a relapse was significantly higher in male participants, those that had advanced fibrosis, and those with baseline HCV RNA of >800,000 IU/mL,^[15]^ whereas Mangia et al proposed that baseline liver elastometry (kPa values), baseline platelets, baseline MELD score, presence of ascites, the presence of esophageal varices, an history of hepatocarcinoma, baseline HCV viremia levels, baseline aspartate transaminase and alanine transaminase values, baseline albumin values, and co-morbidities (such as diabetes and obesity) can be predictors for a treatment response.^[58]^ Degasperi et al also reported independent factors other than IL28B such as cirrhosis, being aged ≥65 years, obesity, IL28B T/T genotype, diabetes, Black race, and high baseline viral load (≥107 IU/mL).^[57]^ Watanabe et al assessed the potential predictive factors associated with SVR-12: they found that independent predictive factors were gender, age, body mass index, baseline values for ALT, White blood cells, platelet counts, total bilirubin, albumin, prothrombin time, α-fetoprotein, HCV-RNA, type-4 collagen, hyaluronic acid, HbA1c, naïve treatment versus re-treatment, RBV adherence, and chronic hepatitis/cirrhosis.^[59]^ The differences between these studies could be explained by the different sample sizes, virus genotypes, clinical situations of the patients, types of patients regarding previous experience to antivirals, and the addition of SOF.

The study had some limitation as it was a case- control one, which means it was retrospective observational study thus limiting studying the effect of genotype polymorphisms on the outcome of DAAs and demonstrating only an association. This affects generalizability of the results. Larger prospective studies are required to confirm the cause and—effect relationship between both variables. Another limitation is that we did not evaluate the effect of IL28B gene polymorphisms on developing different ad-verse effects of DAAs especially liver decompensation and hepatocellular carcinoma and whether IL-12 affected the incidence of adverse effects related to DAAs. We did not evaluate the other extrahepatic effects of DAAs and the association of IL28B and IL-12 to these effects.

## 5. Conclusions

In conclusion, in HCV-4/RNA positive patients we recommend performing genetic analysis for SNPs in IL-28B before initiating antiviral treatment in order to choose the best suitable treatment regimen. We suggest using a SOF--RBV regimen for HCV-4 patients with a C/C variant of IL28B and adding Peg-IFN to SOF--RBV for HCV-4 patients with non-C/C variants of IL28B. Finally, we suggest using baseline IL-12 level as a predictor for treatment failure.

## Author contributions

**Conceptualization:** Doaa Mohamed Abdelnajid.

**Data curation:** Ahmed Y. Elmowafy.

**Formal analysis:** Ahmed Y. Elmowafy.

**Investigation:** Doaa Mohamed Abdelnajid.

**Methodology:** Marwa T. Elrakaiby.

**Project administration:** Doaa Mohamed Abdelnajid.

**Resources:** Doaa Mohamed Abdelnajid.

**Software:** Ahmed Y. Elmowafy.

**Supervision:** Marwa T. Elrakaiby.

**Validation:** Ahmed Y. Elmowafy.

**Visualization:** Doaa Mohamed Abdelnajid.

**Writing – original draft:** Doaa Mohamed Abdelnajid.

**Writing – review & editing:** Lionel Rostaing.
